# Respiratory Support With Nasal High Flow Reduces Opioid Requirements During Endoscopic Retrograde Cholangiopancreatography

**DOI:** 10.1002/deo2.70421

**Published:** 2026-09-04

**Authors:** Tomotaka Mori, Takao Ayuse, Shuntaro Sato, Taiga Ichinomiya, Rintaro Yano, Akane Shimakura, Yasuhiko Nakao, Kosuke Takahashi, Masanori Fukushima, Masafumi Haraguchi, Ryu Sasaki, Eisuke Ozawa, Satoshi Miuma, Stanislav Tatkov, Tetsuya Hara, Hisamitsu Miyaaki

**Affiliations:** ^1^ Department of Gastroenterology and Hepatology Nagasaki University Graduate School of Biomedical Sciences Nagasaki Japan; ^2^ Department of Gastroenterology and Hepatology Japanese Red Cross, Nagasaki Genbaku Hospital Nagasaki Japan; ^3^ Clinical Research Center Nagasaki University Hospital Nagasaki Japan; ^4^ Department of Anesthesiology and Intensive Care Medicine Nagasaki University Graduate School of Biomedical Sciences Nagasaki Japan; ^5^ Fisher & Paykel Healthcare Ltd Auckland New Zealand

**Keywords:** endoscopic retrograde cholangiopancreatography, hypercapnia, intravenous anesthesia, nasal high flow, opioids, randomized controlled trial

## Abstract

**Objectives:**

Nasal high flow (NHF) improves oxygenation, reduces the work of breathing, and stabilizes ventilation. In endoscopic retrograde cholangiopancreatography (ERCP) under dexmedetomidine‐based sedation, opioids are often required but increase the risk of respiratory depression and delayed recovery. This study tested the hypothesis that NHF therapy, compared with low‐flow oxygen (LFO), may reduce opioid requirements during ERCP.

**Methods:**

This open‐label, single‐center, randomized controlled trial enrolled patients undergoing ERCP under dexmedetomidine‐based sedation. Patients were randomized to NHF or LFO. Sedation used dexmedetomidine, midazolam and pethidine with a standardized protocol. The primary endpoint was total pethidine dose. Secondary endpoints included transcutaneous carbon dioxide (TcCO_2_), oxygenation‐perfusion parameters, processed electroencephalography (EEG) parameters, sedative doses, and sedation depth.

**Results:**

Enrollment ended early at a total of 94 of the planned 118 patients due to slow recruitment (NHF 47, LFO 47). Poisson regression demonstrated reduced opioid requirements with NHF (incidence rate ratio 0.77, 95% confidence interval, 0.62–0.96, *p* = 0.023). The total pethidine dose was lower in the NHF group (medians 43.75 mg vs. 70.00 mg, *p* = 0.004). The TcCO_2_ and oxygenation‐perfusion parameters were comparable between groups. EEG tests showed lower patient state index and electromyographic activity in the NHF group, indicating deeper sedation. No adverse events attributable to interventions were observed.

**Conclusion:**

NHF reduced opioid requirements while maintaining oxygenation, ventilation, and sedation during ERCP. The ability of NHF to provide respiratory support, ensure adequate oxygenation, and allow monitoring of breathing makes it a promising modality during procedural sedation; however, further multi‐center studies are needed.

**Trial Registration:**

072210047 [jRCTs]

**Abbreviations**
ARTF%: artifact ratio
AUC: area under the curve
CI: confidence interval
EEG: electroencephalography
EMG: electromyography
ERCP: endoscopic retrograde cholangiopancreatography
FiO_2_
: fraction of inspired oxygen
IRR: incidence rate ratio
LFO: low‐flow oxygen
NHF: nasal high flow
PaCO_2_
: arterial partial pressure of carbon dioxide
PSi: Patient State Index
SEF: spectral edge frequency
SEFL: spectral edge frequency (left)
SEFR: spectral edge frequency (right)
SpO_2_
: peripheral oxygen saturation
SR%: suppression ratio
TcCO_2_
: transcutaneous carbon dioxide

## 1 Introduction

Endoscopic retrograde cholangiopancreatography (ERCP) is a complex endoscopic procedure that often requires deep sedation to ensure patient comfort and procedural success. Sedation during ERCP carries inherent risks, particularly respiratory depression and hypoxemia, which are exacerbated by the use of opioids. Dexmedetomidine has gained attention as a sedative agent because it provides stable hemodynamics [1] and minimal respiratory suppression [2] compared with other sedatives. However, opioids are frequently required as adjuncts to achieve adequate analgesia and sedation, and their use increases the risk of respiratory compromise [3].

Nasal high flow (NHF) oxygen therapy delivers heated and humidified oxygen at high flow rates, reducing dead space ventilation, improving oxygenation, and decreasing the work of breathing [4, 5]. NHF has also been shown to enhance patient comfort and reduce dyspnea perception [5, 6], which may contribute to more stable ventilation during sedation. Previous studies have suggested that NHF may reduce hypoxemia during ERCP, although these investigations primarily focused on oxygenation [7]. Whether NHF can influence ventilation stability or reduce opioid requirements under dexmedetomidine‐based sedation remains unknown. The aim of this randomized controlled trial was to evaluate if NHF reduces opioid requirements compared with low‐flow oxygen (LFO) therapy during ERCP under dexmedetomidine‐based sedation, while maintaining adequate oxygenation and ventilation. The planned sample size was 118 patients (59 per group), based on a priori power calculation using exploratory data on pethidine requirements. Despite several extensions of the study period, recruitment remained challenging, and enrollment concluded with 94 patients.

## 2 Methods

### 2.1 Study Design

This was an open‐label, single‐center, randomized controlled trial involving adult patients undergoing ERCP under sedation. Patients were randomized into NHF and LFO groups.

### 2.2 Intervention

- **NHF Group**: Patients received oxygen via the Airvo 3 (Fisher & Paykel Healthcare, New Zealand) high‐flow nasal cannula system, with an asymmetrical cannula interface, Optiflow + Duet (Fisher & Paykel Healthcare, New Zealand). Humidified air mixed with oxygen was delivered at 50–70 L/min, with the fraction of inspired oxygen (FiO_2_) set at 0.30–0.40. If peripheral oxygen saturation (SpO_2_) fell below 90% during sedation, the FiO_2_ was increased. The attending physician responsible for sedation, who was a gastroenterologist experienced in anesthesia and had previously received sufficient training from a certified anesthesiology training supervisor, selected an NHF rate within the range of 50–70 L/min at their discretion according to each patient's condition during ERCP.
- **LFO Group**: Patients received oxygen through a conventional nasal cannula at 3–5 L/min via a conventional nasal cannula (estimated FiO_2_ 0.32–0.40). If SpO_2_ dropped below 90%, the flow rate was increased up to 5 L/min.

### 2.3 Sedation Protocol

Dexmedetomidine was administered as a loading infusion at 6 µg/kg/h for 10 min. Midazolam (0.05 mg/kg) and pethidine hydrochloride (35 mg) were administered intravenously approximately 5 min after the start of dexmedetomidine loading, according to the predefined protocol. After completing dexmedetomidine loading, the rate was reduced to 0.7 µg/kg/h for maintenance. The sedation depth was titrated to achieve a Ramsay scale of 5–6 [8]. Once patients fell asleep, ERCP was initiated.

If patient movement occurred at the time of scope insertion, a single additional dose of midazolam (0.05 mg/kg) was administered. Thereafter, if body movement was judged to interfere with the performance of ERCP, pethidine hydrochloride was administered intravenously in 17.5 mg increments, in combination with dexmedetomidine, to maintain a Ramsay scale of 5–6.

### 2.4 Endpoints

The primary endpoint was total pethidine dose administered during ERCP. Secondary endpoints included:

- total doses of dexmedetomidine and midazolam.
- transcutaneous carbon dioxide (TcCO_2_) area under the curve (AUC) normalized by the duration from scope insertion to sedation end.
- peripheral oxygenation and perfusion parameters (SpO_2_, heart rate, pleth variability index, and perfusion index).
- respiratory rate.
- processed electroencephalography (EEG) parameters—Patient State Index (PSi), suppression ratio (SR%), electromyography percentage (EMG%), artifact ratio (ARTF%), and spectral edge frequency (SEF Hz).
- sedation depth.
- safety outcomes.

### 2.5 Measurements

- **TcCO_2_**: continuously measured using TCM5 (Radiometer, Denmark). AUC was normalized by the duration from scope insertion to sedation end.
- **Peripheral Oxygenation and Perfusion Parameters**: SpO_2_, pulse rate, pleth variability index, and perfusion index were continuously recorded using Radical‐7 (Masimo, USA). AUC was normalized to the same duration.
- **Respiratory Rate**: continuously recorded using Radical‐7, and in the NHF group, respiratory rate was also recorded by Airvo 3.
- **Processed EEG Parameters**: PSi, EMG%, SR%, ARTF%, and SEF Hz were continuously recorded using SEDLINE. AUC was normalized by the same duration. SEF was calculated as the mean of the left (SEFL) and right (SEFR) SEF values [9, 10].
- **Sedation Depth**: Assessed clinically at regular intervals using the Ramsay scale.
- **Safety**: Blood pressure and adverse events were recorded throughout the procedure.

### 2.6 Procedural Timing Definition

For all continuous physiological measurements—including TcCO_2_, peripheral oxygenation and perfusion parameters (measured using Radical‐7) and processed EEG parameters (measured using SEDLINE)—the analysis window was defined as the duration from scope insertion to sedation end. “Sedation end” was defined as the time point when dexmedetomidine infusion was discontinued at the completion of ERCP.

### 2.7 Sample Size Calculation

The sample size was determined based on the primary endpoint, the total pethidine dose. An exploratory preliminary study showed that the mean total pethidine units (1 unit = 17.5 mg) were 2.774 units in the NHF group and 3.933 units in the LFO group. Assuming that total pethidine units follow a Poisson distribution with mean values of exp(1.02) = 2.774 and exp(1.37) = 3.933, simulations using a Poisson regression model with group as the explanatory variable were performed. With 1000 iterations and a two‐sided significance level of 5%, a minimum of 53 patients per group was required to achieve at least 90% power. Allowing for a 10% dropout rate, the final target sample size was set at 59 patients per group (118 total).

### 2.8 Statistical Analysis

All randomized patients were included in the intention‐to‐treat population. The primary endpoint, the total pethidine dose, was analyzed using a Poisson regression model with group as the explanatory variable. Incidence rate ratios (IRRs), 95% confidence intervals (95% CIs), and two‐sided *p*‐values were calculated, with a significance level of 5%.

For secondary endpoints, continuous variables were compared between groups using the Student's *t*‐test when normally distributed or the Mann–Whitney *U* test when non‐normally distributed. Categorical variables were compared using the chi‐square test or Fisher's exact test, as appropriate. Safety endpoints were summarized descriptively and compared between groups.

Type I error control was applied only to the primary endpoint. Although hypothesis tests were performed for the secondary endpoints also, no adjustment for multiplicity was made, and these results should therefore be interpreted with caution.

Most analyses were performed using R version 4.5.0. Correlation analyses between the respiratory rate measured by Radical‐7 and Airvo 3 were conducted using MATLAB for data processing and GraphPad Prism 10 (GraphPad Software, USA) for statistical testing.

## 3 Results

### 3.1 Patient Enrollment and Baseline Characteristics

A total of 94 patients were enrolled and randomized equally into the NHF (*n* = 47) and LFO (*n* = 47) groups. Baseline characteristics—including age, sex, anthropometric data, and respiratory function—were comparable between groups (Table 1). Baseline laboratory values are shown in Table S1.

**TABLE 1:** Baseline characteristics of patients.

	NHF group (*n* = 47)	LFO group (*n* = 47)
Age, median (range), years	74 (26–84)	71 (48–83)
Male sex, *n* (%)	27 (57.4)	33 (70.2)
Height, median (range), cm	159.5 (143.3–178.6)	162.6 (147.8–177.0)
Weight, median (range), kg	57.9 (37.6–93.3)	56.4 (36.0–82.7)
FEV_1_, median (range), L	2.14 (1.23–4.10)	2.30 (1.07–4.49)
FVC, median (range), L	2.89 (1.83–5.54)	2.94 (1.75–5.32)
FEV_1_/FVC, median (range), %	75.89 (58.21–90.26)	76.21 (47.35–98.86)
Abnormal electrocardiogram, *n* (%)	27 (57.4)	30 (63.8)

FEV_1_, forced expiratory volume in 1 s; FVC, forced vital capacity; LFO, low‐flow oxygen; NHF, nasal high flow.

In the NHF group, flow settings were as follows: 37 patients received 50 L/min, two received 60 L/min, and eight received 70 L/min, with a mean flow rate of 53.8 L/min (standard deviation 7.6).

### 3.2 Patient Flow

**FIGURE 1:**
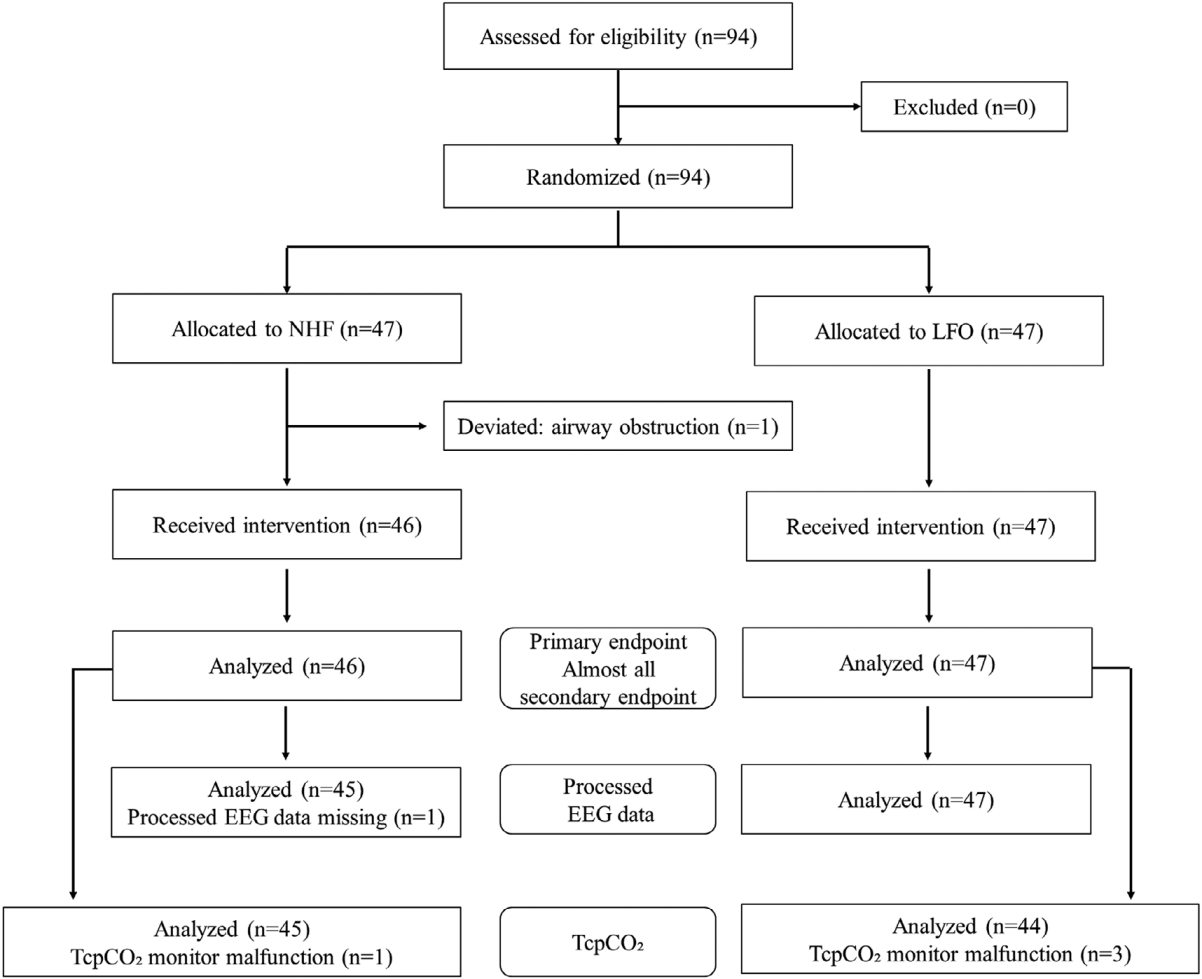
CONSORT flow diagram. Flow of participants through the randomized controlled trial. A total of 94 patients were assessed for eligibility and randomized to NHF (*n* = 47) or LFO (*n* = 47). In the NHF group, 46 received the allocated intervention, and 1 did not due to airway obstruction. During follow‐up, device malfunction or missing physiological monitoring data resulted in reduced evaluable numbers for some secondary endpoints. Primary endpoint analysis included NHF (*n* = 46) and LFO (*n* = 47). Abbreviations: NHF, nasal high flow oxygen; LFO, low‐flow oxygen; EEG, electroencephalography; TcCO_2_, transcutaneous carbon dioxide.

In the NHF group, 46 patients received the allocated intervention; one patient did not receive NHF due to airway obstruction. Device malfunctions or missing data resulted in different numbers of evaluable cases across physiological measurements, including TcCO_2_ monitoring and processed EEG monitoring. The CONSORT diagram is shown in Figure 1 .

**FIGURE 2:**
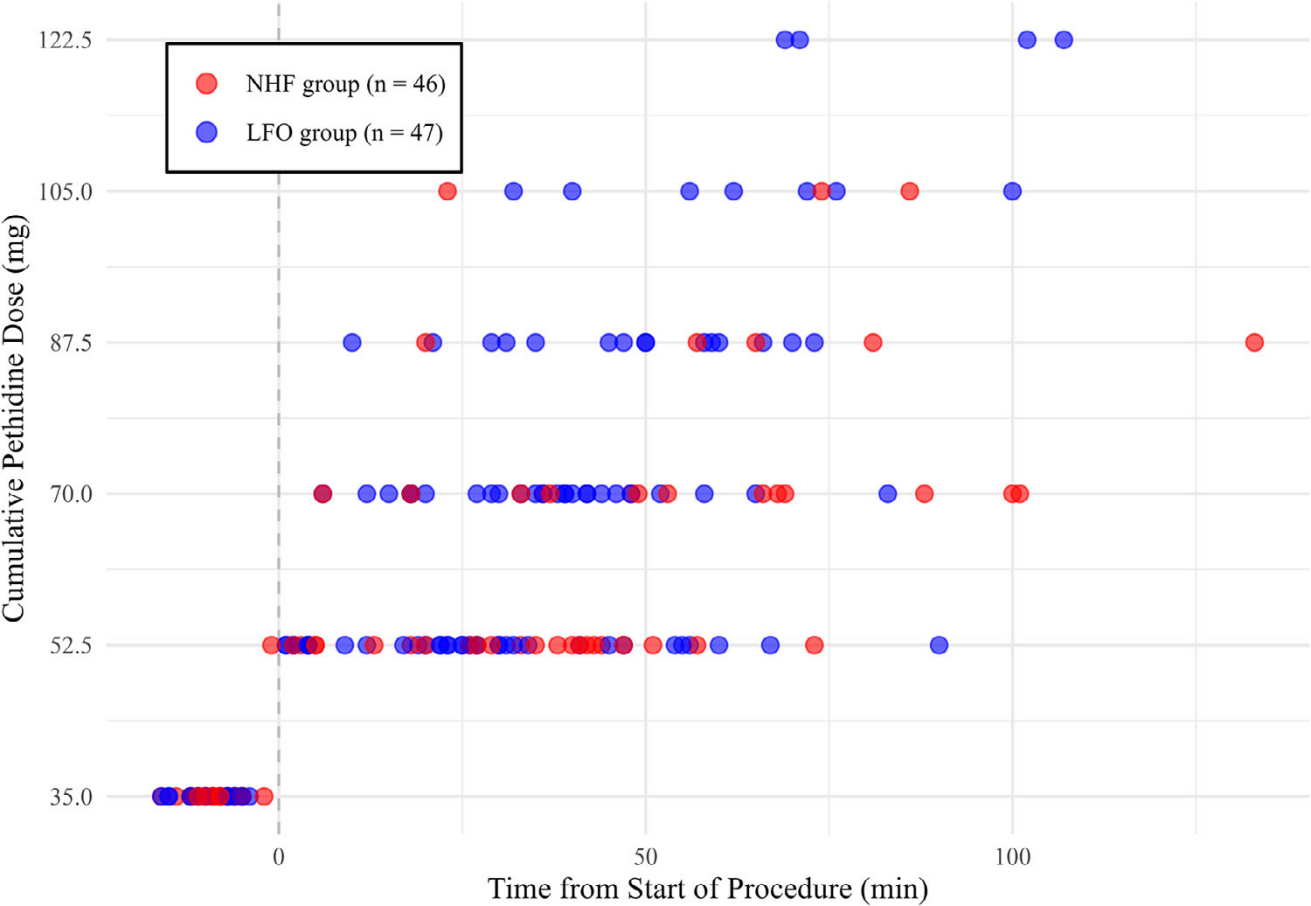
Cumulative Pethidine Administration (Scatter Plot). Each point represents a pethidine dosing event for an individual patient. The cumulative dose (mg) was calculated within each patient by summing all doses administered up to that time point. Time is shown relative to the start of ERCP; negative values reflect initial dosing administered during the dexmedetomidine loading period before the formal start of the procedure. This visualization illustrates the temporal distribution and cumulative trajectory of opioid administration in the NHF and LFO groups. NHF, nasal high flow; LFO, low‐flow oxygen.

### 3.3 Primary Endpoint

The incidence rate of pethidine administration (expressed as 17.5‐mg units per hour of sedation) was lower in the NHF group (2.91 vs 3.77 units/h). We found that NHF was associated with reduced pethidine requirements (IRR 0.77, 95% CI, 0.62 to 0.96, *p* = 0.023). The total pethidine dose was lower in the NHF group (median 43.75 mg vs. 70.00 mg, *p* = 0.004) (Table 2).

**TABLE 2:** Summary of sedation depth, drug use, physiological parameters, and analgesic event rates between groups.

Outcome category	Variable	NHF group	LFO group	Effect estimate	95% CI	*p*‐Value
Analgesic dose	Pethidine units (17.5‐mg units/h)^*^	137/47.0 h (2.91/h)	181/48.1 h (3.77/h)	0.77	0.62–0.96	0.023
	Pethidine total dose (mg)^†^	43.75 (35–105)	70.00 (35–122.50)	—	—	0.004
Sedation depth^†^	Ramsay scale	6.00 (1–6)	6.00 (5–6)	—	—	0.281
	Dexmedetomidine total dose (µg)	96.41 (24.87–220.90)	97.10 (46.50–162.10)	—	—	0.905
	Midazolam total dose (mg)	5.00 (2–10)	5.00 (2–9)	—	—	0.391
Respiratory physiology (AUC)^‡^	TcCO_2_	63.25 ± 13.08	62.07 ± 10.38	1.18	−3.72 to 6.08	0.638
Peripheral oxygenation & perfusion (AUC)^‡^	SpO_2_ (%)	97.19 ± 5.59	98.79 ± 2.35	−1.59	−3.38 to 0.19	0.079
	Pulse rate (bpm)	65.64 ± 11.30	64.79 ± 9.53	0.86	−3.46 to 5.17	0.694
	Respiratory rate (breaths/min)	16.26 ± 3.46	16.25 ± 2.67	0.01	−1.27 to 1.29	0.987
	Pleth variability index	14.68 ± 6.20	15.59 ± 6.79	−0.91	−3.58 to 1.77	0.503
	Perfusion index	3.98 ± 2.60	3.92 ± 2.31	0.07	−0.94 to 1.08	0.892
Processed EEG parameters (AUC)^‡^	Patient State Index	55.69 ± 18.62	65.25 ± 17.33	−9.56	−17.02 to ‐2.10	0.012
	Suppression ratio (%)	0.22 ± 0.49	0.15 ± 0.25	0.07	−0.10 to 0.23	0.412
	Electromyography (%)	13.69 ± 13.83	19.94 ± 14.93	−6.24	−12.20 to ‐0.29	0.040
	Artifact ratio (%)	1.35 ± 2.34	1.24 ± 1.38	0.12	−0.69 to 0.92	0.773
	Spectral edge frequency (Hz)	9.57 ± 4.38	10.79 ± 4.05	−1.22	−2.97 to 0.53	0.170

NHF, nasal high flow; LFO, low‐flow oxygen; TcCO_2_, transcutaneous carbon dioxide.

* Incidence rates were calculated, and the incidence rate ratio with the 95% confidence interval (CI) and *p*‐value were estimated using Poisson regression.

^†^ Data are presented as median (range). Between‐group comparisons were performed using the Wilcoxon rank‐sum test.

^‡^ Data are presented as mean ± standard deviation (SD). Between‐group comparisons were conducted using Welch's t‐test.

### 3.4 Secondary Endpoints

#### 3.4.1 Sedation Depth and Sedative Drug Use

The sedation depth, assessed using the Ramsay scale, did not differ significantly between groups (median 6.00 vs. 6.00, *p* = 0.281). Total doses of dexmedetomidine and midazolam were also comparable between the NHF and LFO groups (*p* = 0.905 and *p* = 0.391, respectively) (Table 2). Figure 2.

#### 3.4.2 Respiratory Physiology

The AUC for TcCO_2_ was similar between groups (means 63.25 vs. 62.07, mean difference 1.18, 95% CI, ‐3.72 to 6.08, *p* = 0.638). No significant differences were observed in the SpO_2_, pulse rate, respiratory rate, pleth variability index, or perfusion index (all *p* > 0.05) (Table 2).

#### 3.4.3 Processed EEG Parameters

The NHF group demonstrated a lower PSi compared with the LFO group (means 55.69 vs. 65.25, mean difference ‐9.56, 95% CI, ‐17.02 to ‐2.10, *p* = 0.012). EMG activity was also lower in the NHF group (mean difference ‐6.24, 95% CI, ‐12.20 to ‐0.29, *p* = 0.040). No significant between‐group differences were observed in the SR%, ARTF%, or SEF (Table 2).

### 3.5 Respiratory Rate Agreement between Airvo 3 and Radical‐7

**FIGURE 3:**
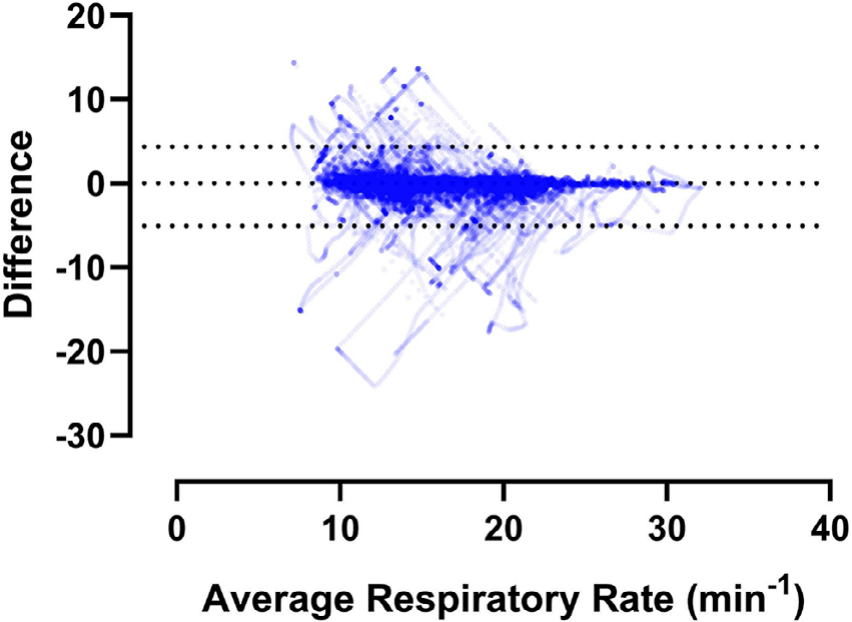
Bland–Altman agreement and correlation between respiratory rates measured by Airvo 3 and Radical‑7. Bland–Altman analysis showed a mean bias of −0.34 breaths/min with 95% limits of agreement −5.06 to 4.37, indicating clinically acceptable agreement between the two devices. Spearman correlation demonstrated a strong association between respiratory rates measured by Airvo 3 and Radical‑7 (*r* = 0.8686, 95% CI 0.8666 to 0.8706, *p* < 0.0001). A total of 63,615 paired measurements were analyzed.

A total of 63,615 simultaneously recorded respiratory‑rate pairs were analyzed. Bland–Altman analysis showed a mean bias of ‐0.34 breaths/min, with 95% limits of agreement from ‐5.06 to 4.37, indicating clinically acceptable agreement between the two devices (Figure 3). Spearman correlation demonstrated a strong association between respiratory rates measured by Airvo 3 and Radical‐7 (*r* = 0.8686, 95% CI, 0.8666 to 0.8706, *p* < 0.0001).

Additional secondary outcomes, including hemodynamic events and complications, are summarized in the Supporting Information. Hemodynamic events and rescue medication use are presented in Table S2. Procedure‑related complications are presented in Table S3.

## 4 Discussion

This randomized controlled trial demonstrated that NHF significantly reduced opioid requirements compared with LFO during ERCP under dexmedetomidine‐based sedation, while maintaining oxygenation and ventilation. Reducing opioid exposure is clinically important because opioids carry dose‐dependent risks of respiratory depression, hypoxemia, and delayed recovery [3]. The present study provides new evidence that NHF not only prevents hypoxemia but also decreases opioid demand during endoscopic procedures.

The mechanisms underlying reduced opioid use with NHF are likely multifactorial. First, NHF decreases the work of breathing, stabilizing ventilation and reducing hypercapnia‐induced arousal [4, 5, 6, 11, 12, 13]. Second, NHF improves patient comfort and reduces dyspnea perception, which helps maintain stable sedation depth [14]. These mechanisms align with physiological studies showing that dyspnea perception activates cortical regions such as the insula and cingulate cortex [15, 16]. By alleviating the respiratory effort and level of discomfort, NHF may indirectly stabilize sedation and reduce opioid demand. These mechanistic explanations represent possible physiological interpretations and remain hypothetical, as they were not directly demonstrated by the present study.

These results align with and extend the findings of the randomized controlled trial by Sawase et al., which compared NHF without supplemental oxygen to LFO during ERCP and found comparable rates of hypoxemia and hypercapnia between the two approaches [7]. A recent multicenter trial, RENOVATE, which compared respiratory support with NHF and noninvasive ventilation in a broad range of patients with acute respiratory failure, revealed that in all groups—except cardiogenic pulmonary edema—fewer patients required dexmedetomidine for sedation [17].

The respiratory threshold for spontaneous breathing during sedation—defined as the apneic threshold on the CO_2_ response curve—is determined by the interaction between sedative drug concentration and comprehensive stress, including pain, dyspnea, and general discomfort [3]. At a given sedative level, weaker stress is associated with a relative elevation of the respiratory threshold, leading to a reduced respiratory rate and increased arterial partial pressure of carbon dioxide (PaCO_2_). In contrast, stronger stress enhances ventilatory drive, resulting in an increased respiratory rate and a relative reduction in PaCO_2_.

Previous studies have demonstrated that NHF reduces respiratory effort and alleviates dyspnea [4]. Therefore, when a balance is achieved between different combinations—such as stress reduction via NHF with low opioid doses versus greater stress under LFO requiring high opioid doses—the respiratory rate and PaCO_2_ may remain at comparable levels despite differences in opioid exposure. In the present study, although the respiratory rate and TcCO_2_ did not differ between the groups, this may reflect titration of pethidine in response to procedure‑interfering movement, which likely equalized perceived procedural stress between groups. This interpretation represents a possible explanation for the observed respiratory parameters and is not presented as a conclusion directly demonstrated by the study. Pethidine was administered as needed in response to patient movement or procedural discomfort, which may have resulted in broadly comparable levels of stress between the groups. This titration strategy may explain the absence of intergroup differences in the respiratory rate and TcCO_2_ AUC.

In addition, PSi values were lower in the NHF group, suggesting deeper sedation [9]. In general, deeper sedation is associated with an upward shift of the apneic threshold on the CO_2_ response curve and reduced ventilatory responsiveness. However, TcCO_2_ remained comparable between groups in this study. This observation may reflect enhanced CO_2_ clearance with NHF [18]. Alternatively, the difference in pethidine dosing between groups may have been insufficient to produce clinically apparent respiratory depression under dexmedetomidine‐based sedation, known to preserve ventilatory drive relative to other sedatives [2].

Furthermore, opioid‐related respiratory effects may have become apparent after the discontinuation of NHF, when the supportive effects of high‐flow therapy were removed. However, postprocedural respiratory measurements were not collected in this study, and further investigation is necessary.

The NHF group exhibited lower PSi and EMG% compared with the LFO group. Lower PSi reflects deeper and more stable sedation depth [9], while reduced EMG% indicates suppression of muscle activity and arousal responses [10]. These findings suggest that NHF enhances sedation stability by reducing respiratory discomfort and minimizing patient movement. Other EEG‐derived indices (SR%, ARTF%, and SEF Hz) did not differ between groups, which indicates that NHF primarily influences arousal‐related components rather than EEG frequency characteristics [10].

NHF reduced pethidine requirements while maintaining comparable respiratory parameters when normalized by sedation duration. The observation that lower opioid exposure coincided with deeper sedation (lower PSi) supports the hypothesis that NHF alleviates respiratory stress and the work of breathing [4, 5, 6, 11, 12]. Although the respiratory rate and TcCO_2_ did not differ between the groups, this may reflect the sedation titration strategy designed to maintain comparable sedation depth, rather than indicating the absence of physiological effects of NHF. Importantly, reducing opioid dosing during ERCP may lower the risk of post‐procedural respiratory depression in the ward setting [3]. Therefore, the stress‐reducing effect of NHF during ERCP carries meaningful clinical significance.

The respiratory rate in the NHF group was measured using two devices. The Radical‐7 uses a microphone attached to the skin with adhesive, whereas the Airvo 3 measures breathing frequency by detecting pressure pulses during NHF via an asymmetrical cannula interface with a large cross‐sectional area of the nasal prongs. Both methods provide continuous monitoring of breathing. The strong correlation with Airvo 3 (*r* = 0.8686) and the narrow Bland–Altman limits of agreement (bias −0.34 breaths/min, limits of agreement −5.06 to 4.37) demonstrate that both devices offer similar respiratory‐rate monitoring under dexmedetomidine‐based sedation (Figure 3).

### 4.1 Limitations

Several limitations should be acknowledged. First, the study was completed with 94 patients, fewer than the planned 118, due to recruitment challenges. This shortfall may have reduced statistical power, particularly for secondary endpoints. Second, the trial was conducted at a single center, which may limit the generalizability of the findings. Third, despite the standardized sedation protocol, physicians’ awareness of group allocation could have influenced the administration of additional pethidine because the study was not blinded. Fourth, flow settings in the NHF group varied (50–70 L/min) according to the attending physician's discretion, which may have introduced heterogeneity. Fifth, the sedation depth was assessed using the Ramsay scale and processed EEG‐derived indices, which may not fully capture patient comfort or subtle fluctuations in the levels of sedation. Finally, long‐term outcomes such as recovery time and patient satisfaction were not evaluated.

## 5 Conclusion

This randomized controlled trial demonstrated that NHF significantly reduced opioid requirements compared with LFO during ERCP performed under dexmedetomidine‐based sedation. NHF with supplemental oxygen maintained adequate oxygenation and ventilation while providing deeper and more stable sedation, as indicated by lower processed EEG‐derived indices and reduced EMG activity. The decreased opioid exposure may help mitigate the risk of post‐procedural respiratory depression in the ward setting. The ability of NHF to provide respiratory support, ensure adequate oxygenation, and allow monitoring of breathing makes it a promising modality during procedural sedation; further multicenter studies are required.

## Author Contributions

**Conception and design**: Takao Ayuse, Shuntaro Sato, Taiga Ichinomiya, Rintaro Yano, Kosuke Takahashi, Masanori Fukushima, Masafumi Haraguchi, Eisuke Ozawa, Stanislav Tatkov, Tetsuya Hara, and Hisamitsu Miyaaki. **Analysis and interpretation of the data**: Tomotaka Mori, Takao Ayuse, Shuntaro Sato, Taiga Ichinomiya, Akane Shimakura, Yasuhiko Nakao, Ryu Sasaki, Satoshi Miuma, and Stanislav Tatkov. **Drafting of the article**: Tomotaka Mori, Shuntaro Sato, Taiga Ichinomiya, and Stanislav Tatkov. **Critical revision of the article for important intellectual content**: all authors. **Final approval of the article**: all authors.

## Conflicts of Interest

Stanislav Tatkov is an employee of Fisher & Paykel Healthcare Ltd., the manufacturer of the nasal high flow devices used in this study. Takao Ayuse received funding and support from Fisher & Paykel Healthcare Ltd. The other authors declare no conflicts of interest.

### Funding

This study was funded by the institutional budget and partly by Fisher & Paykel Healthcare Ltd, Auckland, New Zealand. The Airvo 3 device and related information were provided by Fisher & Paykel Healthcare Ltd.

### Ethics Statement

This study was approved by the Institutional Review Board of Nagasaki University Hospital (approval number: CRB21‐004). All procedures were conducted in accordance with the Declaration of Helsinki.

### Consent

Written informed consent was obtained from all participants.

## Supporting information




**Supporting File**: deo270421‐sup‐0001‐SuppMat.docx.

## References

[1] B. C. Bloor , D. S. Ward , J. P. Belleville , and M. Maze , “Effects of Intravenous Dexmedetomidine in Humans. II. Hemodynamic Changes,” Anesthesiology 77, no. 6 (1992): 1134–1142, 10.1097/00000542-199212000-00014. 1361311

[2] J. P. Belleville , D. S. Ward , B. C. Bloor , and M. Maze , “Effects of Intravenous Dexmedetomidine in Humans. I. Sedation, Ventilation, and Metabolic Rate,” Anesthesiology 77, no. 6 (1992): 1125–1133, 10.1097/00000542-199212000-00013. 1361310

[3] K. T. Pattinson , “Opioids and the Control of Respiration,” British Journal of Anaesthesia 100, no. 6 (2008): 747–758, 10.1093/bja/aen094. 18456641

[4] R. L. Parke and S. P. McGuinness , “Pressures Delivered by Nasal High Flow Oxygen during all Phases of the Respiratory Cycle,” Respiratory Care 58, no. 10 (2013): 1621–1624, 10.4187/respcare.02358. 23513246

[5] J. E. Ritchie , A. B. Williams , C. Gerard , and H. Hockey , “Evaluation of a Humidified Nasal High‐Flow Oxygen System, Using Oxygraphy, Capnography and Measurement of Upper Airway Pressures,” Anaesthesia and Intensive Care 39, no. 6 (2011): 1103–1110, 10.1177/0310057x1103900620. 22165366

[6] J. Bräunlich , M. Köhler , and H. Wirtz , “Nasal Highflow Improves Ventilation in Patients With COPD,” International Journal of Chronic Obstructive Pulmonary Disease 11 (2016): 1077–1085, 10.2147/copd.S104616. PMC4887061 27307723

[7] H. Sawase , E. Ozawa , H. Yano , et al., “Respiratory Support With Nasal High Flow Without Supplemental Oxygen in Patients Undergoing Endoscopic Retrograde Cholangiopancreatography Under Moderate Sedation: A Prospective, Randomized, Single‐Center Clinical Trial,” BMC Anesthesiology 23 (2023):156, 10.1186/s12871-023-02125-w. PMC10165286 37158818

[8] M. A. Ramsay , T. M. Savege , B. R. Simpson , and R. Goodwin , “Controlled Sedation With Alphaxalone‐Alphadolone,” British Medical Journal 2, no. 5920 (1974): 656–659, 10.1136/bmj.2.5920.656. PMC1613102 4835444

[9] D. R. Drover , H. J. Lemmens , E. T. Pierce , et al., “Patient State Index: Titration of Delivery and Recovery From Propofol, Alfentanil, and Nitrous Oxide Anesthesia,” Anesthesiology 97, no. 1 (2002): 82–89, 10.1097/00000542-200207000-00012. 12131107

[10] P. L. Purdon , A. Sampson , K. J. Pavone , and E. N. Brown , “Clinical Electroencephalography for Anesthesiologists: Part I: Background and Basic Signatures,” Anesthesiology 123, no. 4 (2015): 937–960, 10.1097/aln.0000000000000841. PMC4573341 26275092

[11] K. Dysart , T. L. Miller , M. R. Wolfson , and T. H. Shaffer , “Research in High Flow Therapy: Mechanisms of Action,” Respiratory Medicine 103, no. 10 (2009): 1400–1405, 10.1016/j.rmed.2009.04.007. 19467849

[12] J. Bräunlich , D. Beyer , D. Mai , S. Hammerschmidt , H. J. Seyfarth , and H. Wirtz , “Effects of Nasal High Flow on Ventilation in Volunteers, Copd and Idiopathic Pulmonary Fibrosis Patients,” Respiration 85, no. 4 (2013): 319–325, 10.1159/000342027. 23128844

[13] M. Berthon‐Jones and C. E. Sullivan , “Ventilation and Arousal Responses to Hypercapnia in Normal Sleeping Humans,” Journal of Applied Physiology: Respiratory, Environmental and Exercise Physiology 57, no. 1 (1984): 59–67, 10.1152/jappl.1984.57.1.59. 6432751

[14] V. Le Stang , M. Graverot , A. Kimmoun , et al., “Nasal High Flow to Modulate Dyspnea in Orally Intubated Patients,” American Journal of Respiratory and Critical Care Medicine 211, no. 4 (2025): 577–586, 10.1164/rccm.202405-1057OC. 39680951

[15] R. B. Banzett , H. E. Mulnier , K. Murphy , S. D. Rosen , R. J. Wise , and L. Adams , “Breathlessness in Humans Activates Insular Cortex,” Neuroreport 11, no. 10 (2000): 2117–2120, 10.1097/00001756-200007140-00012. 10923655

[16] R. W. Lansing , R. H. Gracely , and R. B. Banzett , “The Multiple Dimensions of Dyspnea: Review and Hypotheses,” Respiratory Physiology & Neurobiology 167, no. 1 (2009): 53–60, 10.1016/j.resp.2008.07.012. PMC2763422 18706531

[17] Investigators, RENOVATE, and the BRICNet Authors . “High‐Flow Nasal Oxygen vs Noninvasive Ventilation in Patients With Acute Respiratory Failure: The Renovate Randomized Clinical Trial,” JAMA 333, no. 10 (2025): 875–890, 10.1001/jama.2024.26244. PMC11897836 39657981

[18] T. Mauri , L. Alban , C. Turrini , et al., “Optimum Support by High‐Flow Nasal Cannula in Acute Hypoxemic Respiratory Failure: Effects of Increasing Flow Rates,” Intensive Care Medicine 43, no. 10 (2017): 1453–1463, 10.1007/s00134-017-4890-1. 28762180

