# Correction to “Respiratory Support with Nasal High Flow Reduces Opioid Requirements During Endoscopic Retrograde Cholangiopancreatography”

**DOI:** 10.1002/deo2.70442

**Published:** 2026-09-23

T. Mori, T. Ayuse, S. Sato, et al., “Respiratory Support With Nasal High Flow Reduces Opioid Requirements During Endoscopic Retrograde Cholangiopancreatography,” *DEN Open* 7, no. 1 (2027): e70421, https://doi.org/10.1002/deo2.70421.

There was an error in the author list for the above article. The author name “Taiga Ichinomia” was incorrect and should have read “Taiga Ichinomiya.” The online article has been corrected.

We apologize for this error.

