# Supplementary material for: Respiratory Support With Nasal High Flow Reduces Opioid Requirements During Endoscopic Retrograde Cholangiopancreatography
Source: DEN Open. 2026 Sep 4;7(1):e70421. doi: 10.1002/deo2.70421 (PMC13542827; doi:10.1002/deo2.70421)
Supplement: Supplementary file 1 [file DEO2-7-e70421-s001.docx]

| Supplementary Table 1. Baseline characteristics of patients | | |
| --- | --- | --- |
|  | NHF group | LFO group |
| WBC, median (range), ×10³/μL | 5.70 (1.10–12.00) | 5.90 (1.60–10.40) |
| RBC, median (range), ×10⁶/μL | 4.22 (2.17–5.88) | 4.15 (2.98–4.99) |
| Hb, median (range), g/dL | 13.20 (7.20–17.80) | 12.70 (8.40–16.10) |
| Hct, median (range), % | 39.10 (23.00–54.30) | 38.10 (27.00–46.90) |
| Plt, median (range), ×10³/μL | 226.00 (62.00–563.00) | 218.00 (114.00–460.00) |
| ALP, median (range), U/L | 110.00 (46.00–1876.00) | 158.00 (52.00–957.00) |
| AST, median (range), U/L | 24.00 (10.00–833.00) | 38.00 (12.00–442.00) |
| ALT, median (range), U/L | 28.00 (7.00–842.00) | 51.00 (9.00–583.00) |
| LDH, median (range), U/L | 170.00 (103.00–394.00) | 191.00 (103.00–287.00) |
| γ-GTP, median (range), U/L | 94.00 (12.00–2559.00) | 223.00 (13.00–2502.00) |
| T-Bil, median (range), mg/dL | 0.90 (0.40–28.70) | 0.90 (0.30–24.40) |
| D-Bil, median (range), mg/dL | 0.30 (0.10–21.80) | 0.30 (0.00–17.10) |
| TP, median (range), g/dL | 7.20 (5.50–8.20) | 7.20 (6.00–8.30) |
| Alb, median (range), g/dL | 3.70 (2.20–4.80) | 3.70 (2.50–4.40) |
| BUN, median (range), mg/dL | 13.00 (4.00–29.00) | 14.00 (5.00–32.00) |
| Tcho, median (range), mg/dL | 179.00 (0.51–672.00) | 191.00 (0.35–640.00) |
| Amy, median (range), U/L | 77.00 (26.00–875.00) | 75.00 (28.00–1621.00) |
| P-Amy, median (range), U/L | 33.00 (6.00–866.00) | 30.00 (4.00–1608.00) |
| CRP, median (range), mg/dL | 0.21 (0.00–7.36) | 0.31 (0.02–15.51) |
| NHF, nasal high flow; LFO, low‑flow oxygen; WBC, white blood cell count; RBC, red blood cell count; Hb, hemoglobin; Hct, hematocrit; Plt, platelet count; ALP, alkaline phosphatase; AST, aspartate aminotransferase; ALT, alanine aminotransferase; LDH, lactate dehydrogenase; γ‑GTP, gamma‑glutamyl transpeptidase; T‑Bil, total bilirubin; D‑Bil, direct bilirubin; TP, total protein; Alb, albumin; BUN, blood urea nitrogen; Tcho, total cholesterol; Amy, amylase; P‑Amy, pancreatic amylase; CRP, C-reactive protein. | | |

| Supplementary Table 2. Hemodynamic events and rescue medication use between groups | | | |
| --- | --- | --- | --- |
| Outcome | NHF group | LFO group | *p*-value |
| SBP ≤ 90 mmHg | 25 (54.3%) | 26 (55.3%) | 1.000 |
| MAP ≤ 65 mmHg | 18 (39.1%) | 22 (46.8%) | 0.532 |
| SBP drop ≥ 40 mmHg | 27 (58.7%) | 24 (51.1%) | 0.534 |
| SBP ≥ 160 mmHg | 14 (30.4%) | 14 (29.8%) | 1.000 |
| SBP increase ≥ 20% | 10 (21.7%) | 15 (31.9%) | 0.351 |
| Ephedrine use | 9 (19.6%) | 11 (23.4%) | 0.802 |
| Nicardipine use | 1 (2.2%) | 0 (0.0%) | 0.495 |
| Naloxone use | 2 (4.3%) | 5 (10.6%) | 0.435 |
| Flumazenil use | 0 (0.0%) | 4 (8.5%) | 0.117 |
| NHF, nasal high flow; LFO, low-flow oxygen; SBP, systolic blood pressure; MAP, mean arterial pressure. | | | |

| Supplementary Table 3. Complications (CTCAE grade and procedure‑related incidence) | | | |
| --- | --- | --- | --- |
| Category |  | NHF group | LFO group |
| CTCAE grade | |  |  |
| Any grade |  | 12 (25.5%) | 14 (29.8%) |
| Grade 4 |  | 1 (2.1%) | 2 (4.3%) |
| Grade 3 |  | 6 (12.8%) | 5 (10.6%) |
| Grade 2 |  | 5 (10.6%) | 4 (8.5%) |
| Grade 1 |  | 2 (4.3%) | 3 (6.4%) |
| Event name | |  |  |
| Acute pancreatitis | | 7 (14.9%) | 4 (8.5%) |
| Acute cholangitis | | 3 (6.4%) | 3 (6.4%) |
| Abdominal pain | | 1 (2.1%) | 5 (10.6%) |
| Oxygen desaturation | | 1 (2.1%) | 1 (2.1%) |
| Duodenal perforation | | 0 (0%) | 1 (2.1%) |
| Acute liver injury | | 1 (2.1%) | 0 (0%) |
| Urticaria |  | 1 (2.1%) | 0 (0%) |
| Extravasation at injection site | | 0 (0%) | 1 (2.1%) |
| Fever |  | 0 (0%) | 1 (2.1%) |
| NHF, nasal high flow; LFO, low-flow oxygen; CTCAE, Common Terminology Criteria for Adverse Events. | | | |
